# Supplementary material for: Modelling Skylarks (Alauda arvensis) to Predict Impacts of Changes in Land Management and Policy: Development and Testing of an Agent-Based Model
Source: PLoS One. 2013 Jun 6;8(6):e65803. doi: 10.1371/journal.pone.0065803 (PMC3675089; doi:10.1371/journal.pone.0065803)
Supplement: Supporting Information S4 — The skylark ODdox as a zipped archive. (ZIP) [file pone.0065803.s004.zip › Skylark_ODdox/elements_8h.html]

ALMaSS Skylark ODdox: elements.h File Reference


ALMaSS Skylark ODdox
 2.0


- Main Page
- Related Pages
- Classes
- Files

- File List
- File Members

Classes |
Macros |
Typedefs |
Functions |
Variables

elements.h File Reference

|  |  |
| --- | --- |
| Classes | |
| class | ActivePit |
| class | AmenityGrass |
| class | BareRock |
| class | BeetleBank |
| class | Building |
| class | BuiltUpWithParkland |
| class | Coast |
| class | ConiferousForest |
| class | Copse |
| class | DeciduousForest |
| class | Field |
| class | FieldBoundary |
| class | ForestElement |
| class | Freshwater |
| class | Garden |
| class | GreenElement |
| class | Heath |
| class | HedgeBank |
| class | Hedges |
| class | LargeRoad |
| class | LE |
| class | LE\_TypeClass |
| class | Marsh |
| class | MixedForest |
| class | NaturalGrass |
| class | NonVegElement |
| class | Orchard |
| class | OrchardBand |
| class | OrchardGrass |
| class | Parkland |
| class | PermanentSetaside |
| class | PermPasture |
| class | PermPastureLowYield |
| class | PermPastureTussocky |
| class | PitDisused |
| class | Railway |
| class | River |
| class | RiversidePlants |
| class | RiversideTrees |
| class | RoadsideVerge |
| class | RuralResidential |
| class | Saltwater |
| class | SandDune |
| class | Scrub |
| class | SmallRoad |
| class | StoneWall |
| class | Suburban |
| class | Track |
| class | UnsprayedFieldMargin |
| class | Urban |
| class | UrbanNoVeg |
| class | UrbanPark |
| class | VegElement |
| class | YoungForest |

|  |  |
| --- | --- |
| Macros | |
| #define | EL\_HERBICIDE\_DELAYTIME   (g\_el\_herbicide\_delaytime\_days.value()) |
| #define | EL\_MAX\_USERSPACE   10 |
| #define | EL\_STRIGLING\_DELAYTIME   (g\_el\_strigling\_delaytime\_days.value()) |
| #define | EL\_TRAMLINE\_DECAYTIME   (g\_el\_tramline\_decaytime\_days.value()) |
| #define | LE\_SIG\_NO\_FUNGICIDE   0x0001 |
| #define | LE\_SIG\_NO\_GROWTH\_REG   0x0008 |
| #define | LE\_SIG\_NO\_HERBICIDE   0x0020 |
| #define | LE\_SIG\_NO\_INSECTICIDE   0x0002 |
| #define | LE\_SIG\_NO\_MOLLUSC   0x0010 |
| #define | LE\_SIG\_NO\_SYNG\_INSECT   0x0004 |

|  |  |
| --- | --- |
| Typedefs | |
| typedef unsigned int | LE\_Signal |

|  |  |
| --- | --- |
| Functions | |
| static CfgStr | l\_el\_bug\_percentage\_file ("ELEM\_BUG\_PERCENTAGE\_FILE", CFG\_CUSTOM,"bugpercents.txt") |
| static CfgBool | l\_el\_read\_bug\_percentage\_file ("ELEM\_READ\_BUG\_PERCENTAGE\_FILE", CFG\_CUSTOM, false) |

|  |  |
| --- | --- |
| Variables | |
| CfgInt | g\_el\_herbicide\_delaytime\_days |
| CfgInt | g\_el\_strigling\_delaytime\_days |
| CfgInt | g\_el\_tramline\_decaytime\_days |
| class Landscape \* | g\_landscape\_p |
| class LE\_TypeClass \* | g\_letype |

---

## Macro Definition Documentation

#define EL\_HERBICIDE\_DELAYTIME   (g\_el\_herbicide\_delaytime\_days.value())

Referenced by Farm::HerbicideTreat(), and Farm::Trial\_PesticideTreat\_GS().

#define EL\_MAX\_USERSPACE   10

#define EL\_STRIGLING\_DELAYTIME   (g\_el\_strigling\_delaytime\_days.value())

Referenced by Farm::Strigling(), and Farm::StriglingSow().

#define EL\_TRAMLINE\_DECAYTIME   (g\_el\_tramline\_decaytime\_days.value())

Referenced by Farm::BurnStrawStubble(), Farm::CutToHay(), Farm::CutToSilage(), Farm::CutWeeds(), Farm::FA\_AmmoniumSulphate(), Farm::FA\_GreenManure(), Farm::FA\_Manure(), Farm::FA\_NPK(), Farm::FA\_PK(), Farm::FA\_Sludge(), Farm::FA\_Slurry(), Farm::FP\_GreenManure(), Farm::FP\_LiquidNH3(), Farm::FP\_ManganeseSulphate(), Farm::FP\_Manure(), Farm::FP\_NPK(), Farm::FP\_NPKS(), Farm::FP\_PK(), Farm::FP\_Sludge(), Farm::FP\_Slurry(), Farm::FungicideTreat(), Farm::GrowthRegulator(), Farm::Harvest(), Farm::HayBailing(), Farm::HayTurning(), Farm::HerbicideTreat(), Farm::HillingUp(), Farm::InsecticideTreat(), Farm::Molluscicide(), Farm::ProductApplication0(), Farm::ProductApplication1(), Farm::RowCultivation(), Farm::StrawChopping(), Farm::Strigling(), Farm::StriglingSow(), Farm::Swathing(), Farm::SynInsecticideTreat(), Farm::Trial\_Control(), Farm::Trial\_PesticideTreat(), Farm::Trial\_PesticideTreat\_GS(), and Farm::Trial\_ToxicControl().

#define LE\_SIG\_NO\_FUNGICIDE   0x0001

Referenced by Farm::FungicideTreat().

#define LE\_SIG\_NO\_GROWTH\_REG   0x0008

Referenced by Farm::GrowthRegulator().

#define LE\_SIG\_NO\_HERBICIDE   0x0020

Referenced by Farm::HerbicideTreat().

#define LE\_SIG\_NO\_INSECTICIDE   0x0002

Referenced by Farm::InsecticideTreat().

#define LE\_SIG\_NO\_MOLLUSC   0x0010

Referenced by Farm::Molluscicide().

#define LE\_SIG\_NO\_SYNG\_INSECT   0x0004

Referenced by Farm::SynInsecticideTreat().

---

## Typedef Documentation

typedef unsigned int LE\_Signal

---

## Function Documentation

|  |  |  |  |
| --- | --- | --- | --- |
| static CfgStr l\_el\_bug\_percentage\_file | ( | "ELEM\_BUG\_PERCENTAGE\_FILE" | , |
|  |  | CFG\_CUSTOM | , |
|  |  | "bugpercents.txt" |  |
|  | ) |  |  |

static

Referenced by VegElement::ReadBugPercentageFile().

|  |  |  |  |
| --- | --- | --- | --- |
| static CfgBool l\_el\_read\_bug\_percentage\_file | ( | "ELEM\_READ\_BUG\_PERCENTAGE\_FILE" | , |
|  |  | CFG\_CUSTOM | , |
|  |  | false |  |
|  | ) |  |  |

static

Referenced by VegElement::VegElement().

---

## Variable Documentation

CfgInt g\_el\_herbicide\_delaytime\_days

CfgInt g\_el\_strigling\_delaytime\_days

CfgInt g\_el\_tramline\_decaytime\_days

class Landscape\* g\_landscape\_p

Referenced by Farm::AutumnHarrow(), Farm::AutumnPlough(), Farm::AutumnRoll(), Farm::AutumnSow(), Farm::BurnStrawStubble(), Farm::CattleIsOut(), Farm::CattleIsOutLow(), Farm::CattleOut(), Farm::CattleOutLowGrazing(), Farm::CutToHay(), Farm::CutToSilage(), Farm::CutWeeds(), Farm::DeepPlough(), VegElement::DoDevelopment(), Field::DoDevelopment(), Orchard::DoDevelopment(), OrchardBand::DoDevelopment(), Farm::FA\_AmmoniumSulphate(), Farm::FA\_GreenManure(), Farm::FA\_Manure(), Farm::FA\_NPK(), Farm::FA\_PK(), Farm::FA\_Sludge(), Farm::FA\_Slurry(), Farm::FP\_GreenManure(), Farm::FP\_LiquidNH3(), Farm::FP\_ManganeseSulphate(), Farm::FP\_Manure(), Farm::FP\_NPK(), Farm::FP\_NPKS(), Farm::FP\_PK(), Farm::FP\_Sludge(), Farm::FP\_Slurry(), Farm::Harvest(), Farm::HayBailing(), Farm::HayTurning(), Farm::HillingUp(), Farm::PigsAreOut(), Farm::PigsAreOutForced(), Farm::PigsOut(), VegElement::RecalculateBugsNStuff(), Farm::RowCultivation(), Farm::SleepAllDay(), Farm::SpringHarrow(), Farm::SpringPlough(), Farm::SpringRoll(), Farm::SpringSow(), Farm::StrawChopping(), Farm::Strigling(), Farm::StriglingSow(), Farm::StubbleHarrowing(), Farm::Swathing(), Farm::Water(), and Farm::WinterPlough().

class LE\_TypeClass\* g\_letype

Referenced by Landscape::BackTranslateEleTypes(), Landscape::BackTranslateVegTypes(), Farm::HandleEvents(), Farm::InitiateManagement(), Landscape::Landscape(), Landscape::ReadPolys(), Landscape::TranslateEleTypes(), Landscape::TranslateVegTypes(), and Landscape::~Landscape().


- CJT
- MSVC
- ALMaSS Working Source
- Landscape
- elements.h
- Generated on Thu Jan 10 2013 13:15:35 for ALMaSS Skylark ODdox by
   1.8.1.1
